# The OPERA trial: protocol for a randomised trial of an exercise intervention for older people in residential and nursing accommodation

**DOI:** 10.1186/1745-6215-12-27

**Published:** 2011-02-02

**Authors:** Martin Underwood, Sandra Eldridge, Sallie Lamb, Rachel Potter, Bartley Sheehan, Anne-Marie Slowther, Stephanie Taylor, Margaret Thorogood, Scott Weich

**Affiliations:** 1 Health Sciences Research Institute, Warwick Medical School, University of Warwick, Coventry CV4 7AL, UK; 2 Centre for Health Sciences, Queen Mary University of London, , 2 Newark Street, London E1 2AT, UK

## Abstract

**Abstract:**

**Background**

Depression is common in residents of Residential and Nursing homes (RNHs). It is usually undetected and often undertreated. Depression is associated with poor outcomes including increased morbidity and mortality. Exercise has potential to improve depression, and has been shown in existing trials to improve outcomes among younger and older people. Existing evidence comes from trials that are short, underpowered and not from RNH settings. The aim of the OPERA trial is to establish whether exercise is effective in reducing the prevalence of depression among older RNH residents.

**Method**

OPERA is a cluster randomised controlled trial. RNHs are randomised to one of two groups with interventions lasting 12 months

**Intervention group**: a depression awareness and physical activity training session for care home staff, plus a whole home physical activation programme including twice weekly physiotherapist-led exercise groups. The intervention lasts for one year from randomisation, or

**Control group**: a depression awareness training session for care home staff.

Participants are people aged 65 or over who are free of severe cognitive impairment and willing to participate in the study. Our primary outcome is the prevalence of depressive symptoms, a GDS-15 score of five or more, in all participants at the end of the one year intervention period. Our secondary depression outcomes include remission of depressive symptoms and change in GDS-15 scores in those with depressive symptoms prior to randomisation. Other secondary outcomes include, fear of falling, mobility, fractures, pain, cognition, costs and health related quality of life. We aimed to randomise 77 RNHs.

**Discussion**

Home recruitment was completed in May 2010; 78 homes have been randomised. Follow up will finish in May 2011 and results will be available late 2011.

**Trial Registration:**

[ISRCTN: ISRCTN43769277]

## Background

Untreated depression is one major cause of morbidity in older people, particularly in those who live in residential and nursing homes (RNHs). Up to 40% of RNH residents are depressed [1,2]. The annual incidence of depression is around 12%, with depression resolving after a year in only about half of cases [3]. In many cases the depression is not recognized by the RNH staff or by the resident's general practitioner [4,5].

There is a move away from drug treatment for mild/moderate depression. Guidelines from the UK National Institute of Health and Clinical Excellence (NICE) do not recommend drug treatment for mild depression and suggest that drugs should be used only as part of a more holistic package of care for those with moderate depression [5]. The conventional medical approach of opportunistic diagnosis and drug treatment is insufficient to address depression in this population because of poor recognition, low intervention rates and the toxicity of medications. In care homes tricyclic agents appeared effective but poorly tolerated [4], whilst more modern drugs are well tolerated but are less well researched in this setting [6,7]. The use of drugs as the primary approach to managing depression in these patients presents three problems: the failure to recognise mild/moderate depression, the absence of evidence of effectiveness in the very elderly (aged > 80), [8] and the potential for serious adverse events, in particular amongst those on multiple medications. Typically RNH residents are on 6-8 different drugs, with most receiving at least one psychotropic agent [1,9]. Conditions such as cardiac disease, diabetes, and renal impairment that are likely to require caution in prescribing antidepressants among older people are highly prevalent in RNH residents (32%, 20% & 4% respectively) [10]. Falls are a major cause of morbidity and mortality in older people and antidepressant drugs; both tricyclics and SSRIs are associated with a two-fold increase in falls in care home residents [11].

Multiple physical morbidities are expected in RNH residents [12] and lack of social interaction has been shown to be common in observational research in residential settings [3]. There is good evidence that both functional impairment and loneliness are risk factors for depression in RNH residents [12]. There is a growing recognition that addressing single outcomes such as falls, cardiovascular disease or depression as isolated problems inadequately addresses the needs of this group[13]. Depression is a viable target for public health interventions as physical health and costs of care are significantly higher for older people with depression [14,15].

Exercise is a promising non-medical approach to the management of depression. Plausible mechanisms for its possible effect include improved social contact, a diversion from negative thoughts, and the physiological effects on neurotransmitters such as monoamines & endorphins [16]. Experimental evidence also supports progressive strength training as a method of reducing depression [17].

A number of systematic reviews which pooled data from different study designs found that exercise improved depression [15,18]. A review of 14 randomised controlled trials suggested that exercise might be efficacious in reducing symptoms of depression in the short term, although all of these studies had significant limitations. No statistically significant differences were found between aerobic and anaerobic exercise regimens [15]. NICE guidelines recommend exercise as a first line treatment for mild depression [19]. A systematic review of the effects of exercise on depression in older people concluded that it might be efficient at reducing depressive symptoms in the short term but that there were insufficient data on its long-term effect [18]. Interventions were of short duration, the trials were small, none were in RNH settings and none sought to change participants' approach to exercise throughout the week [18].

Maximising psychological and physiological effects from an exercise regimen requires that increased exercise is built into residents' usual routine. However active residents may be during brief formal exercise sessions, these will occupy only a small percentage of their time. Engineering a system change within RNHs so that increased exercise is both facilitated and actively encouraged will ensure that residents are regularly exposed to the intervention throughout the week. In this trial we are testing a pragmatic intervention, reflective of current best practice, consisting of training for RNH staff to support the building of safe physical activity into the RNHs' normal routine; and a twice-weekly formal exercise class led by a specially trained physiotherapist. This is a 'whole RNH' intervention; all residents without an absolute contraindication to exercise will be invited to attend the class and to increase physical activity generally. This allows depressed residents to benefit from interaction with non-depressed residents; and reflects how such a programme, if effective, might be implemented in RNHs by physiotherapists. Furthermore, it may: avoid the use of drug treatment; demedicalise mild/moderate depression; confer wider health benefits; improve social interactions within RNHs; and have beneficial effects on all residents.

The protocol reported here was developed for an NHS Health Technology Assessment (HTA) funded trial and amended after a pilot study to test the procedures [20]. A process evaluation was commissioned to run alongside this trial and is reported separately [21].

## Methods

### Research aim

To establish the impact of a 'whole home' intervention, consisting of training for residential and nursing home staff combined with twice-weekly evidence based, physiotherapist-led exercise class, on the prevalence of depression amongst residents.

### Trial design

This is a cluster-randomised trial with the RNH as the unit of randomisation and residents as the unit of assessment to measure the impact of a whole RNH intervention to increase exercise on the prevalence of depression and the remission of existing depression. To reduce NHS treatment costs we are using an unbalanced randomisation:- 1 intervention home:1.5 control homes.

### Sample size

The original brief from the NHS HTA programme specified the outcome as rate of remission in those depressed at baseline. We estimated that we needed to recruit 77 RNHs and at least 418 residents with depression at baseline to detect an increase in remission rates from 25% to 40%. Our primary analysis will be a comparison of the difference in proportion of depressed residents at the end of the study (40% v 25%). Recruiting participants from 77 RNHs gives more than 97% power to detect this at the 5% significance level, even if we need to exclude residents recruited post-randomisation from this comparison. Our sample size estimate includes an inflation factor to account for clustering effects. Few previous studies are available to allow us to estimate the range of likely values for the intra-cluster correlation (ICC) needed to estimate this. We therefore used a conservative value of 0.05 for this, which towards the upper end of the range seen in previous primary care studies. This should provide have sufficient power to allow for likely variation in cluster sizes and for likely clustering effects due to different physiotherapists carrying out the exercise programme in different RNHs.

Our calculations also allow for anticipated loss to follow-up. Because few RNH residents move out of residential accommodation we anticipate good follow-up rates. This population has a high mortality, up to 34% per year; additionally, for some, their health will deteriorate so that they are no longer able to complete some, if not all, of the follow-up assessments. However, those residents with the poorest health are less likely to join the study, so that we can anticipate a smaller attrition rate. The nearest equivalent study collected data on 169/220 depressed residents after 9.5 months (77%) [22], equivalent to 71% at one year. We therefore anticipate a loss to follow-up rate of 30%, made up of those who have died and those no longer willing or able to complete the assessments.

### Homes and Participants recruitment

Recruitment of both the home and residents within the home at recruitment takes place prior to randomisation of the RNH to the intervention or control arm. We are recruiting roughly equal numbers of RNHs in two localities, North East London and the West Midlands. These two localities have diverse populations that are representative of the social and ethnic mix of the UK as a whole. We are seeking to recruit RNHs with a range of characteristics (large/small, independent/chain, local authority/private/charitable, purpose-built/traditional, nursing/residential). One pilot home and one home recruited early in the study were dementia specialist homes, but we found that the proportion of residents able to complete the questions for our primary outcome measure (the geriatric depression scale - 15 item version) was small and concluded that recruiting such homes was not the best use of our resources for recruitment and intervention

#### Eligibility Criteria

We have set our entry criteria for our assessments as wide as possible to ensure generalisability of our results. The study is open to any older person living in a residential or nursing home who is able to communicate and is fit enough to participate in the assessments (Table 1). To contribute to our depression outcomes participants need to be able to provide GDS-15 data.

**Table 1:** Inclusion and exclusion criteria

Inclusion Criteria	Exclusion criteria
Assessments	

Permanent resident in RNH	Problems communicating by any means
Aged 65 or over	Terminal or other serious illness
Consent/assent to assessment	Non-English speakers for who a translator is not available
Able to participate in baseline assessment	

Consent/assent to record examination	

#### Recruiting Participants

We consider for inclusion all permanent residents in RNHs, unless staff of the RNH identify a resident as inappropriate to approach for consent/assent either directly or via their next of kin, for example those with a very limited life expectancy. A specially trained health professional briefly assesses each individual to explain the study, to assess his/her competence to give consent and to participate in the research assessments, and assess where he/she meets the inclusion criteria (Table 1). Residents are given information about the study in a suitable format (e.g. large print or audio). The staff of the RNH together with research nurse identify residents who are unable to participate in any assessments but who nevertheless may be able to give consent to the use of their routine data. This group might include those unable for any reason to communicate in English sufficiently well to participate in the assessments. Competent residents have the opportunity to reflect on whether they wish to join the study prior to giving consent to the assessments and/or the use of their medical records. The next of kin of those deemed not competent to consent to the study assessments are approached for their assent for the resident to take part in the assessments and/or the use of their medical records. We repeat the recruitment procedure for any new permanent residents in participating RHNs up to nine months following that RNH's randomisation.

### Baseline assessments and data collection

Baseline assessments, lasting around 30-60 minutes, are carried out by a research nurse and take place after consent/assent has been obtained and before randomisation. At this assessment, we confirm eligibility, administer the questionnaire instruments (GDS-15, EuroQol, MMSE, fear of falling, current pain) and a brief physical assessment, the Short Physical Performance Battery (SPPB). We collect demographic data (age, sex, ethnicity, social class and age left school) and data on length of residence, fee status and current medication from the RNH records for all those for whom we have consent/assent to access their records. We collect data for the Barthel Index, social engagement scale, and proxy values for the EQ-5D from the care staff.

The diagnosis of depression is commonly overlooked in RNH residents [22]. It would be inappropriate, once we have identified depression, to prevent these residents from accessing appropriate health care, so we provide the RNH staff with participants' Geriatric Depression Score (GDS-15) scores, and an interpretation of their meaning. If the GDS score is 10 or more we encourage the home to discuss this result with the resident's general practitioner.

### Data management

All data are managed in line with Warwick Clinical Trial Unit's standard operating procedures.

### Randomisation

For each location, we minimise by size and type of home. Homes are categorized by size and by a combination of ownership (for profit/charitable/local authority, ) and services offered (residential/nursing/both). A statistician separate from the rest of the study team is responsible for generating the homes' random allocation using a computerised minimisation program.

A particular problem for cluster randomised trial can be the need to recruit participants after the cluster has been randomised. For our primary outcome, prevalence of depression one year after randomisation may be affected by this since we are including participants who moved into the homes following randomisation. For these residents randomisation allocation concealment is impossible because all RNH staff and study staff visiting the RNHs are aware of the home's allocation [23,24]. We ensure that we are aware of all new residents and monitor reasons for exclusion from the study. For our secondary outcomes related to improvement in depressive symptoms we only include participants resident in the homes at the start of the study. All of their baseline data are collected prior to randomisation to ensure that for this analysis we can achieve allocation concealment.

### Interventions

#### Control Intervention

In line with the MRC's Good Clinical Practice Guidelines there is an active control intervention in all participating RNHs to ensure that they are aware of current best care for the identification and management of depression in this population. This intervention consists of depression awareness training for staff in the RNH. By using current best care as our control, we can ensure that any benefits identified are due specifically to our intervention package rather than to raising awareness of depression within the intervention RNHs. This intervention reduces the risk of 'resentful demoralisation' in the control homes affecting recruitment of new residents after randomisation or even leading to the RNH withdrawing from the study [25].

The depression awareness programme consists of brief (around 45-60 minutes) training backed up with a specially created DVD on recognizing depression and information leaflets and posters. In addition we inform the staff of all RNHs of the GDS-15 scores of all participating residents. In the control homes our research nurses will deliver this intervention soon after randomisation. In the active intervention RNHs the depression awareness training will be delivered by the research physiotherapists as part of the overall intervention package. More details of the control intervention package are available on request from the research team.

#### Active intervention

We aim to test a pragmatic intervention, which could be implemented as part of routine health/social care. This is a 'whole-home' intervention aiming to change the culture of the home to increase promotion of physical activity. The intervention includes a **physical activation programme**, supported by a twice-weekly **physiotherapist-led exercise class**. The intervention will continue for one year in each home. We anticipate that our intervention, as well as improving mood should also improve functional abilities, reduce falls, and improve mobility.

Several studies have found that low intensity and poorly implemented physical activation programmes may increase falling [26,27] and pose a threat to the safety of older people living in nursing homes. We consider it essential to the safety of participants that experienced therapists implement the intervention. They need to have the essential awareness of the multiple health problems facing older people in care settings, of exercise prescription in frail older adults, and of promoting safe mobility.

Therapists attend a two-day course to be trained in the intervention. The intervention is documented and standardised, but allows therapists a range of options to adapt the content to the needs of the groups/settings. This approach has been successful in previous studies we have conducted with physiotherapists; therapists are happy that the approach reflects their practice and, importantly, it is possible to document the concept and method with the precision necessary for replication and dissemination [28]. This approach utilises recognised methods of selecting baseline exercise intensity, and progressing exercise [17] modified for frail older people. Exercise that utilises rhythmic movement and reflex motor activation is effective in people with cognitive impairment [29]. The intervention seeks to minimise unwanted side-effects including an increase in falls and pain.

The physical activation programme promotes a commitment to encouraging safe physical activity for residents of the home. Evidence suggest that for a complex intervention of this nature to be effective it needs to be 'normalised'; that is, it should be seen as part of the organisation's normal functioning rather than as an optional extra [30]. If a positive approach to increasing exercise in the residents is built into the values of RNH staff, the likelihood of an exercise intervention having a positive effect will be maximised. The main activity that is targeted in the physical activation programme is safe walking, which is a realistic aim [31].

A growing body of evidence on diffusion of innovation and implementation of guidelines into clinical practice has highlighted organisational context as a key factor in effecting change in practice [32,33]. Successful implementation is more likely if the intervention/guideline resonates with the experience of those being asked to embrace it [32,34] Embedding the exercise intervention within a whole-home strategy that actively engages with the organisational context in which the intervention is delivered should enhance the effectiveness of implementation.

The physiotherapists:

1. Improve knowledge and awareness of the benefits of physical activity in the staff, residents and relatives.

2. Provide an individualised review of mobility safety.

3. Ensure that appropriate and safe walking aids (including grab rails where needed), and footwear are available to each individual, and reinforce the need for use[35].

4. Provide advice on activation strategies for individual clients, including the level/type of assistance/supervision needed, and support the staff and residents in their implementation.

5. With the Director of Nursing/Home Manager, review the policies and strategies in place to promote physical activity.

6. Where appropriate, involve volunteers and families in the supervision and promotion of physical activity.

All residents are invited to attend twice-weekly **group-based exercise **classes in the communal space of the homes. All new residents are encouraged to enter the groups as they enter the home. An exercise intervention as part of routine care in a residential setting should be available to all residents, not just those with diagnosed depression. This is both for the practical reason that it removes the requirement to formally to identify those entitled to attend and also because such an intervention it is less likely to be effective if only those who are depressed attend.

The groups are led by physiotherapists experienced in managing frail older people.The sessions use mixed training stimuli, combining aerobic conditioning, progressive strength and balance training. The programme utilises music and rhythmic, simple movement patterns. The groups have around 12 to 15 participants, and last 40 minutes to an hour depending on the tolerance and ability of the group. Prior to the groups, the physiotherapists give each participant a brief risk assessment; determining any absolute contra-indications to exercise, and the optimal exercise intensity for each participant. The physiotherapist may have a group with a range of abilities, and thus intensity is set to ensure safety of all participants. In larger homes, where there may be a need for several groups, physiotherapists group together participants with similar levels of ability, and set the intensity of exercise accordingly. It is challenging to gain engagement and attendance from depressed individuals, but maximising participation is essential to demonstrating effectiveness of the technology. Consent/assent to study assessments does not indicate consent to participate in the exercise class on any particular day. We comply with best clinical practice for consent to physiotherapy treatment to obtain agreement from individuals for participation in the exercise class. The physiotherapists work with staff to gain participants' trust and acceptance of the programme.

### Follow-up

Follow-up assessments for all consenting/assented residents take place six months and one year after the home is randomised. Data from medical records are collected 3-monthly. If any residents have moved to another RNH in the locality when their assessments are due, we endeavour to carry out their assessments in these alternative locations.

### Outcome measures

The **primary outcome measure **is the geriatric depression scale 15 (GDS-15) [36]. This brief scale/score consists of 15 simple yes/no questions and has been well validated in residential situations. It avoids using potentially somatic features of depression which may be misleading in this age group, focusing more on mood and functional symptoms of depression[37]. It is one of the most widely used measures in this field. It is simple to complete, with 97% of cognitively intact nursing home residents producing analysable data, and it has good internal consistency in this population, and a score of five or above appears to give the best sensitivity and specificity [36-38]. The GDS-15 has also been used as a continuous measure in at least one RCT based in a nursing home[39].

All residents will be exposed to the intervention and any positive or negative effects may impact both those who are depressed and those who are not depressed. We will therefore measure outcomes in all participants. With a long term (one year) intervention we expect the effects to accrue, and for this reason our primary outcome is depression in residents at one year follow-up. Because we expect some change in the population during the year due to deaths and other movements out of the homes, we have chosen to base our primary outcome on residents in the home at twelve months, rather than those residents at baseline.

We had also planned to collect data at six months on those participants with depressive symptoms GDS-15 ≥5 at baseline. During the first few months of recruitment the average cluster size was smaller than originally anticipated, (around 11 residents per home rather than 16), reducing our statistical power. With the agreement of the independent Trial Steering Committee we changed to collecting data on all participants at six months allowing us to also test the mean change in number of depressive symptoms. We selected six months because if exercise were an effective treatment for depression we would expect a response in a similar time to that of drugs. In drug trials we would expect maximal effect to be seen by four months, but there will be a time lag between randomisation and any benefits from the exercise programme because of the time taken to start changing the attitudes of the RNH staff and to establish the class as a regular routine in the RNH. We estimate that the intervention is fully functional two months after randomisation; and thus our first assessment is at six months.

Because we want to understand as much as possible about how the intervention works in practice and to explore any barriers to implementation we have included a number of secondary outcome measures:

#### Depression

Change in severity of depressive symptoms in depressed residents (GDS-15) and proportion of residents depressed at baseline that experience remission from that depression (GDS-15).

#### Cognition

Mini-mental State examination (MMSE) [40]. This scale allows a reliable assessment of degree of cognitive impairment. Sensitive to change and reliable in multiple care settings, it is the most commonly used scale to assess degree of cognitive impairment.

#### Health-related quality of life

EuroQol is a 5-item generic scale with a validated health utility index for calculation of QALYs (Quality Adjusted Life Years) [41]. It has been successfully used in care home populations [42].

#### Physical function

To assess the effect of the programme on mobility we will use the Short Physical Performance Battery (SPPB), which incorporates three essential aspects of mobility that should be improved by the exercise programme; balance, chair rise and walking ability. The SPPB has been used extensively in trials and observational studies of older people. Because of the central importance of physical function to the ability to thrive, has well established and surprisingly strong relationships with a range of important public health outcomes, including onset and progression of disability; mortality and nursing home admission [43,44].

#### Chronic Pain

We will ask participants to rate their current level of pain, i.e. pain today, on a five-point numerical rating scale at each follow-up

#### Fear of falling

We will ask participants about their fear of falling (yes/no); this is an independent predictor of falls risk [45].

#### Mortality

We will arrange for the records of all those from whom we obtain consent/assent to examine their records to be flagged at NHS central registry.

#### Hospital admissions

We will extract data on cause and duration of any hospital admissions during the study period from participants' hospital records.

#### Peripheral fractures

Rather than measuring falls, most of which do not cause injury, we will measure the rate of peripheral fractures as a marker for injurious falls. We will identify these from the RNH and hospital records.

#### Medication use

We will estimate participants' medication use over the follow-up period using data on their regular medications collected at baseline and subsequent time points. We will use these data to estimate their total use of medication over the study period.

#### Cost-effectiveness

We will assess the cost-effectiveness of the exercise programme from both a societal and an NHS perspective.

### Statistical analysis

We will compare the characteristics of RNHs (location, type and size of home) and individuals (age, sex, baseline assessment scores) in our intervention and control arms using simple descriptive statistics. We will summarise outcome measures by RNH and, in the intervention arm, physiotherapist, and use computer software that can measure the extent of clustering at two levels to estimate clustering parameters.

To assess the difference in proportions depressed at 12 months between intervention and control homes we will initially use generalised estimating equations (to account for clustering by RNH) with a logit link (to allow for a binary outcome) in Stata. For other outcomes we will use different link functions as appropriate. We will include covariates in our analysis. Ideally these should be chosen on the basis of predictive value. As far as possible we will assess evidence from the previous literature to identify potential covariates. However, the strategy of choosing covariates based on predictive value arises from work pertaining to individually randomised trials and application to cluster randomised trials is more complicated, particularly for individual level covariates and binary outcomes [46,47]. In addition, the previous literature from which we can identify cluster-level covariates is not extensive. We therefore propose the following strategies for covariate identification:

For our primary outcome, the proportion of residents depressed at the end of the study, we will consider the following cluster-level variables for incorporation as covariates in our models:

• Location of home (our stratification variable).

• Measurable characteristics of care homes and residents identified from previous literature as related to the prevalence of depression;

• Baseline level of depression in care home (based on empirical evidence that baseline levels of outcome are often important covariates);

• Other measurable characteristics of care homes or residents within care homes hypothesised by the study team as being related to outcome.

The covariates to be included will be finalised in a statistical analysis plan prior to analysts becoming un-blinded to randomisation group.

For other outcomes: change in severity of depression for those depressed at baseline, remission rate and geriatric depression score-15 score we will use a similar strategy, but for these outcomes measurements at baseline and outcome will be of the same individual. We will therefore introduce baseline levels of depression severity as covariates at the individual, rather than the cluster, level.

If our preliminary analyses indicate the presence of additional clustering by physiotherapist, we will need to incorporate this in our analyses of outcomes. This will be done using WinBUGs software which allows the building of flexible mixed-effect models such as that needed to incorporate clustering by physiotherapist, which if it exists, will exist in only one arm of our trial.

### Health economic analysis

Our primary analysis will be a cost utility analysis examining the cost per quality adjusted life year gained for all those residents we have assessed. We will not have health utility data on those who did not participate in the assessments. These residents will also have been exposed to the intervention. We will therefore do a secondary costs and consequences analysis to assess the impact of our programme on the prescribing and other health care costs for all residents where we have consent/assent to examine their records. The costs of the exercise programme to RNHs, in terms of time taken to set up and monitor the exercise routines, or to implement the control intervention, will be estimated from data provided by participating RNHs. Other important resource units to capture are prescription costs; GP and, for residential homes, community nurse consultations; attendance at hospital A&E and outpatients; and hospital bed-days. These health service usage data will be collected from RNH and hospital medical records. We will use the EuroQol to calculate health utility for our cost-utility analysis.

### Ethical considerations

This study is a complex intervention within a cluster-randomised trial involving a potentially vulnerable participant population, some of whom will be unable to give consent. Thus raises a number of ethical issues, including consent to cluster randomisation, individual consent to the exercise component of the intervention, participation in research assessments and access to records. Specific measures have been incorporated into the study to address these issues, drawing on recommendations for good practice as well as legal and regulatory frameworks [46,48-50]. To ensure compliance with Mental Capacity Act 2005 sections 3, 30-34 we seek next of kin assent for those residents not competent to consent[51]. The process evaluation study for this project includes observation of the consent process and exploration of residents and next of kin views on this.

To explore issues of consent further we will, hold two focus groups to explore the views of key informants in the wider community (representatives from local and national user groups) about carrying out this study in nursing and residential homes and the process of consent for such research. This will inform ethical analysis of our approach to recruitment and consent.

The Joint University College London/University College London Hospital Committees on the Ethics of Human Research. Reference number 07/Q0505/56 provided ethical review for the study

### Adverse Event Monitoring

We have prepared a protocol for monitoring and reporting adverse events. The protocol covers the specific requirements for recording and reporting adverse events in a cluster randomised trial where adverse events are expected to be high due to the age and frailty of the participants. The protocol distinguishes between adverse events that are directly attributable to the study interventions, and the monitoring of peripheral fractures and deaths indirectly attributable to the study. The trial has a Data Monitoring and Ethics Committee

## Discussion

At the time of writing, April 2010, over 70 homes have been randomized with an average number of residents providing GDS-15 data of around 11 per home. It is anticipated that recruitment will be completed in May 2010. Follow up will be completed one year later and the results available late 2011. Although it is not possible to comment on the effectiveness of the intervention it is evident that both interventions are well received within the homes and the exercise groups are popular with the residents.

## Abbreviations

A&E: Accident and Emergency; GDS: Geriatric Depression Scale; GP: General Practitioner; HTA: Health Technology Assessment; ICC: Intra-Cluster Correlation; MMSE: Mini Mental State Examination; MRC: Medical Research Council; NHS: National Health service; NICE: National Institute for Health and Clinical Excellence; OPERA: Older Peoples Exercise in Residential and nursing Accommodation; RCT: Randomised Controlled Trial; RNH: residential or nursing home; SPPB: Short Physical performance Battery; TSC: Trial Steering Committee; UK: United Kingdom;

## Competing interests

BS has received a conference travel grant from Janssen, and spoken at meetings supported by the pharmaceutical industry. None of the other authors have any competing interests

## Authors' contributions

MU is principal investigator, led the successful application for funding, assembled the trial team, led the ethics application, and all revisions of the protocol and the generation of this protocol paper. SE conducted sample size calculations and designed the study analysis SL contributed to the development of the research protocol and has led the design and management of the physical activation/exercise intervention. She has contributed to the management of the project. RP led the recruitment of RNHs and residents. BS led the development of the control intervention (depression awareness programme), and assisted the recruitment strategy for RNHs. AS advises on ethical issues related to the study and will provide ethical analysis of the consent process. ST leads delivery of the London arm of the study. MT leads the process evaluation and wrote the first draft of this paper. All the authors contributed to the design of the trial protocol, read and commented on drafts and approved the final manuscript.
